# Proteinuria as an Unusual Presentation of Pheochromocytoma in Pediatric Patient With Single Kidney

**DOI:** 10.1155/crpe/1761397

**Published:** 2026-09-24

**Authors:** Jacob Miller, Nycole Hidalgo, Maria Eleni Nikita, Teresa York, Bakri Alzarka

**Affiliations:** ^1^ University of Maryland Medical Center, Baltimore, Maryland, USA, umaryland.edu; ^2^ University of Maryland School of Medicine, Baltimore, Maryland, USA, umaryland.edu; ^3^ Department of Pediatrics, University of Maryland School of Medicine, Baltimore, Maryland, USA, umaryland.edu

**Keywords:** hypertension, pediatrics, pheochromocytoma, proteinuria, single kidney

## Abstract

Pheochromocytoma is a rare catecholamine‐secreting tumor, particularly in pediatric populations, and often presents with sustained hypertension. We report the case of a 17‐year‐old male with a solitary kidney who presented with asymptomatic hypertension and unexpected nephrotic‐range proteinuria. Imaging and biochemical workup confirmed a right adrenal pheochromocytoma. The patient underwent a successful laparoscopic adrenalectomy, after which his blood pressure normalized and proteinuria resolved. This case highlights a rare presentation of pheochromocytoma with heavy proteinuria, likely related to glomerular hyperfiltration secondary to hypertension and exacerbated by a solitary kidney. This case underscores the importance of considering pheochromocytoma in pediatric patients with unexplained hypertension, even in the presence of significant proteinuria.

## 1. Introduction

Pheochromocytomas are rare catecholamine‐ secreting tumors derived from chromaffin cells in the adrenal medulla. Although these tumors are mostly benign, their excessive sympathetic activation can precipitate life‐threatening symptoms. Surgical resection after appropriate medical preparation is the treatment. The classic presentation is hypertension with a triad of headache, sweating, and palpitations, although they can be asymptomatic or paroxysmal [1]. Pheochromocytomas are especially rare in pediatric populations, accounting for only 10%–20% of cases, and the broad range of presentations in these patients complicates diagnosis [1]. Compared to adults who mostly present with the classic triad in paroxysmal attacks, pediatric and adolescent patients tend to exhibit sustained hypertension as a presenting symptom in the majority of cases [2].

A single kidney detected in pediatric patients can represent unilateral renal agenesis or a completely involuted multicystic dysplastic kidney (MCDK). Nephrotic range or heavy proteinuria is defined by urine protein excretion of ≥ 1000 mg/m^2^ per day. Heavy proteinuria is not known to be associated with pheochromocytoma or a single kidney. We report an unusual case of pheochromocytoma in a 17‐year‐old boy with a single kidney presenting with asymptomatic hypertension and nephrotic‐range proteinuria.

## 2. Case Description

This is a 17‐year‐old male presenting with incidentally detected high blood pressure at home and confirmed at 170/110 mmHg at presentation to the emergency department. He was otherwise asymptomatic, though review of systems later identified increased sweating.

His past medical history is notable for chronic kidney disease secondary to a single right kidney detected at 3 years old during a several‐month hospital stay for bilateral streptococcal empyemas and purulent endocarditis, with multiple complications including becoming tracheostomy‐ and gastrostomy‐dependent, though both devices were removed over a decade prior to this presentation. He had otherwise been growing and developing normally without a known diagnosis of hypertension. His family history was negative for hypertension or neuroendocrine tumors.

His physical exam was notable for obesity, hypertension, and intermittent tachycardia. He was otherwise well‐appearing, well‐developed, with no edema or any skin findings to suggest a diagnosis known to be associated with increased risk of developing pheochromocytoma.

### 2.1. Diagnostic Assessment

**FIGURE 1:**
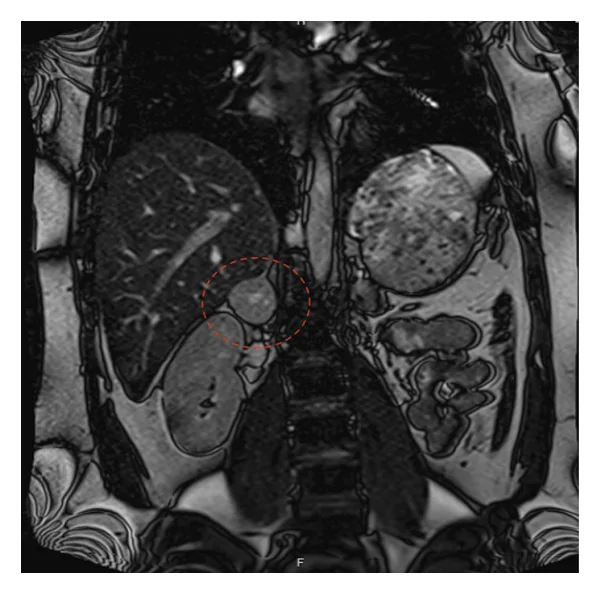
Coronal T2‐weighted magnetic resonance image of the abdomen and pelvis demonstrating a well‐circumscribed, rounded right adrenal mass (red dashed circle) with heterogeneous signal intensity, with central areas of necrosis and small foci of hemorrhage, consistent with likely pheochromocytoma. The left renal fossa is empty, confirming a solitary right kidney with normal corticomedullary differentiation. The liver and spleen appear unremarkable.

His initial laboratory work‐up, including complete blood count, complete metabolic panel including serum creatinine at 0.9 mg/dL (79.6 μmol/L) and serum albumin at 4.3 g/dL (43 g/L), D‐dimer, thyroid‐stimulating hormone, Pro‐BNP, and troponin, were unremarkable. However, urinalysis revealed 4+ proteinuria. A CT angiogram of the chest, abdomen, and pelvis was obtained, demonstrating a right adrenal mass (Figure 1). He was admitted to the hospital for further evaluation and management, and a multidisciplinary team was assembled, including pediatric nephrology, oncology, endocrinology, and genetics.

Based on the findings above, a comprehensive evaluation was initiated. Aldosterone was within normal limits, reducing the likelihood of an aldosterone‐secreting adenoma; plasma renin was mildly elevated, making monogenic hypertension syndromes less likely. A dexamethasone suppression test was normal, as were his plasma testosterone, estradiol, dehydroepiandrosterone sulfate (DHEAS), and 17‐hydroxyprogesterone, making adrenocortical carcinoma an unlikely culprit.

Complementary imaging workup included an echocardiogram showing no evidence of coarctation of the aorta or cardiac end‐organ damage such as left ventricular hypotrophy. Doppler ultrasonography of the renal vasculature and CT angiogram of the abdomen and pelvis showed no evidence of mid‐aortic syndrome or renal artery stenosis. The right kidney is normal structurally and measured at 14.8 cm in length, consistent with expected compensatory hypertrophy in the setting of a single kidney.

A 24‐h urine collection showed a urine protein excretion of 7500 mg/day or 3400 mg/m^2^/day, consistent with nephrotic‐range proteinuria; a random urine sample collected at presentation showed a urine protein to creatinine ratio of 3.2 mg/mg of creatinine. Urinalysis was not suggestive of urinary tract infection, with negative leukocyte esterase and nitrites, and repeated urine microscopy demonstrating no white blood cells or bacteria.

An abdominal MRI with and without contrast revealed a heterogeneously enhancing mass measuring 4.1 × 3 cm (Figure 1). The mass showed mixed signal intensity with central areas of necrosis and small foci of hemorrhage, consistent with likely pheochromocytoma. Importantly, the absence of a signal drop on “out‐of‐phase” images suggested that a fat‐containing lesion, such as an adrenal adenoma or lipoma, was less likely.

**FIGURE 2:**
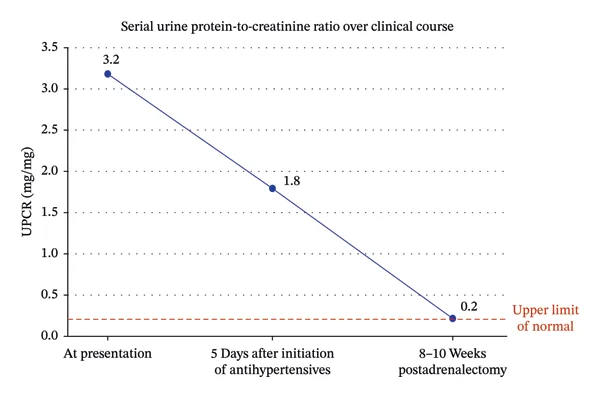
Serial urine protein‐to‐creatinine ratio (UPCR) over the clinical course. UPCR decreased from 3.2 mg/mg at presentation to 1.8 mg/mg five days after initiation of antihypertensive therapy, and further normalized to 0.2 mg/mg at 8–10 weeks following adrenalectomy. The dashed horizontal line represents the upper limit of normal (0.2 mg/mg).

Notably, plasma chromogranin A level was significantly elevated at 521 ng/mL, suggesting a neuroendocrine tumor. Plasma‐free normetanephrine and 24‐h urine normetanephrine and norepinephrine were elevated, clinching a biochemical diagnosis of pheochromocytoma. Plasma‐free metanephrine and 24‐h urine metanephrine and dopamine were within normal limits (Table 1), suggesting this was mainly a norepinephrine‐secreting tumor. Clinical exome sequencing was performed using a paired‐end next‐generation sequencing platform, with a mean coverage of 120x and 98.2% of target regions covered at ≥10x, with variant interpretation guided by the patient’s phenotype. No pathogenic or likely pathogenic variants associated with adrenal mass, hypertension, or chronic kidney disease were identified. This result does not completely exclude a genetic etiology, as variants in noncoding regions or genes not yet associated with disease may not be detected by exome sequencing. His asymptomatic hypertension was initially treated with amlodipine, a calcium channel blocker, while undergoing evaluation. Once the diagnosis was established, phenoxybenzamine was initiated for alpha‐adrenergic blockade. The dose was progressively escalated to achieve adequate blockade, as assessed by target preoperative blood pressure and heart rate. Interestingly, his proteinuria improved with better blood pressure control; a repeat random urine sample was at 1.8 mg/mg of creatinine (Figure 2). The patient underwent laparoscopic right adrenalectomy, which he tolerated well. There was no evidence of adrenal insufficiency, suggesting normal left adrenal gland function.

**TABLE 1:** Biochemical profile.

Analyte	Preoperative (×ULN)	Postoperative (8–10 wk)	Reference range
24‐h urine normetanephrine (μg/24 h)	8792 (15.9)	327	82–553
24‐h urine metanephrine (μg/24 h)	134	77	56–289
24‐h urine norepinephrine (μg/24 h)	1873 (19.5)	—	12–96
24‐h urine dopamine (μg/24 h)	435		100–496
Plasma free normetanephrine (nmol/L)	13.75 (15.9)	0.43	0–0.89
Plasma free metanephrine (nmol/L)	0.11	< 0.10	0–0.49
Chromogranin A (ng/mL)	521 (2.8)	—	0–187
Urine creatinine (mg/kg/24 h)‐ (collection adequacy)	21	—	15–25
Urine vanillylmandelic acid/Cr ratio (mg/g)	10 (1.1)	—	0–9
Renin activity (ng/mL/h)	6 (1.5)	—	0.5–4
Aldosterone (ng/dL)	18.2	—	4–31

*Note:* ×ULN = times upper limit of normal; VMA = vanillylmandelic acid; Cr = creatinine; “—” = not measured. Preoperative values were obtained at initial diagnostic workup. Postoperative values were obtained 8–10 weeks after right laparoscopic adrenalectomy to assess for biochemical cure.

The resected tumor measured 4.5 cm at its biggest dimension, and histopathological examination confirmed the diagnosis of pheochromocytoma with a Pheochromocytoma of the Adrenal Gland Scaled Score (PASS) score of 0. Postoperatively, the patient’s blood pressure improved without the need for antihypertensives, and his proteinuria resolved. At 8–10 weeks postoperatively, repeat urine and plasma catecholamines were normal, suggestive of remission without recurrence or metastasis. A formal ophthalmologic evaluation to assess for hypertensive retinal changes was not performed during the patient’s hospital stay and represents a limitation of this report.

## 3. Discussion

Estimates of the prevalence of the classic pheochromocytoma triad of headache, sweating, and palpitations range from 17%–57% of cases, though pediatric data are underrepresented in the literature [1, 2]. As the dearth of pediatric data would suggest, these tumors are exceedingly rare in the pediatric population. Among children, epidemiologic data suggest the median age at presentation is 11–13 years. Sustained hypertension as a presenting symptom is more common in pediatric patients, though one case series suggests paroxysmal hypertension is rare in adult populations as well [2, 3]. Pheochromocytoma is classically associated with genetic syndromes including Von Hippel–Lindau (VHL), Neurofibromatosis 1 (NF1), and Multiple endocrine neoplasia 2 (MEN2). Patients with genetic predisposition are more likely to present with bilateral, metastatic disease [4, 5]. Accordingly, genetic testing is recommended for all patients. In this patient, no pathogenic germline variant was identified on clinical exome sequencing; however, this does not exclude a genetic etiology given the limitations of current testing and the ongoing discovery of novel susceptibility genes. Importantly, recurrence has been reported even in apparently sporadic cases. These findings underscore the need for long‐term biochemical and imaging surveillance regardless of genetic testing results.

Based on a literature review, typical presenting symptoms included headache, palpitations, and sweating associated with unexplained hypertension [1, 2]. Unfortunately, many patients present with complications of hypertensive emergency and catecholamine surge, such as visual symptoms and retinopathy [6–8], and neurologic complications such as posterior reversible encephalopathy syndrome and stroke [9, 10]. Cardiac complications including cardiomyopathy and myocarditis are also reported [11, 12].

A single kidney is usually related to unilateral renal agenesis or involution of a MCDK. A kidney ultrasound performed in the neonatal period can help differentiate, as most MCDK completely involute between 1 and 3 years of age [13]. Reviewing the literature, there is no known association between pheochromocytoma and chronic kidney disease.

Proteinuria is not typically associated with pheochromocytoma. Despite nephrotic‐range proteinuria detected in our patient on presentation, there was no edema or significant hypoalbuminemia to suggest nephrotic syndrome. The etiology of proteinuria is not completely understood. Previous case reports showed nephrotic‐range proteinuria in patients with pheochromocytoma similar to our case [14, 15], with hypertension and proteinuria resolving with tumor resection as well. The authors suggested that proteinuria etiology is related to glomerular hyperfiltration secondary to hypertension or immunological pathogenesis. It is not clear from the previous case reports if proteinuria improved with blood pressure control before surgical resection. In this current case, patient’s proteinuria improved to the non‐nephrotic range with blood pressure control even before surgical resection, suggesting glomerular hyperfiltration as the more likely etiology. Hypertension can lead to glomerular hyperfiltration, which is defined as an increase in the glomerular filtration rate (GFR) in multiple ways, including but not limited to increasing intraglomerular pressure, vascular remodeling, and activation of the renin–angiotensin–aldosterone system (RAAS) [16].

The single kidney diagnosis in our case probably contributed to a higher degree of glomerular hyperfiltration and proteinuria. Patients with a solitary kidney are at higher risk of relative glomerular hyperfiltration, similar to other conditions associated with a decreased number of functional nephrons such as prematurity. The glomerular filtration rate in each of the remaining nephrons is increased [17], with glomerular hyperfiltration leading to a glomerular mechanical stress injury, podocyte injury, and increased proteinuria [18].

The significant improvement in patient’s proteinuria with better blood pressure control makes immunological pathogenesis less likely. Proteinuria and nephrotic syndrome secondary to malignancy, such as in secondary membranous nephropathy, is a known phenomenon; however, the exact mechanism is not known. The most well‐established mechanism is immune complex deposition, with tumor antigens deposited in the glomerulus and stimulating antibody production, leading to in situ or circulating immune complex formation, and complement activation leading to glomerular basement membrane damage and related proteinuria [19]. In addition, tumors may secrete circulating permeability factors that directly injure podocytes; for example, lung cancer cell–secreted proteins have been shown to activate focal adhesion kinase (FAK) signaling in podocytes and cause foot process effacement [20]. Further research is needed to gain a deeper understanding of the potential immunological effects of pheochromocytoma and the underlying mechanisms of associated proteinuria.

The presence of nephrotic‐range proteinuria in patients with hypertension usually suggests glomerular disease as the etiology of hypertension and may require a kidney biopsy to further evaluate. However, kidney biopsy is generally approached with caution in patients with a solitary kidney due to the higher risk of complications. This case, in conjunction with previous reports, suggests that the diagnosis of pheochromocytoma should be considered, even in the presence of heavy proteinuria, making its diagnosis more challenging. The resolution of proteinuria and hypertension after surgical resection of pheochromocytoma suggests that an early kidney biopsy for the evaluation of nephrotic‐range proteinuria may be avoidable if pheochromocytoma is considered as the underlying cause.

Data on the optimal perioperative blood pressure control in pediatric pheochromocytoma are limited; the therapeutic goals are to lower blood pressure and achieve symptom resolution. Alpha‐adrenergic blockade is typically initiated first, followed by beta‐adrenergic blockade if needed to control tachycardia. It is a well‐established principle that alpha‐adrenergic blockade must be initiated prior to beta‐blockade in the management of pheochromocytoma. Administering a beta‐blocker in the absence of adequate alpha‐blockade eliminates beta‐2–mediated vasodilation in peripheral blood vessels, leaving alpha‐1‐adrenergic vasoconstriction unopposed. This may precipitate a severe hypertensive crisis due to catecholamine‐driven vasoconstriction. Calcium channel blockers have been used in adults with pheochromocytoma successfully [21]. We used amlodipine, a calcium channel blocker, to control our patient’s blood pressure while undergoing evaluation successfully.

Overall, the strength of our approach to this case was the early and rapid mobilization of a multidisciplinary team. This allowed for efficient and definitive management of the patient’s pheochromocytoma before the development of complications, which are, unfortunately, a common presentation in the literature. While our patient’s presenting symptoms are typical of a pheochromocytoma, the presence of nephrotic‐range proteinuria is a rare presentation with only a limited number of cases reported in the literature. In this report, we expanded on the possible mechanisms of proteinuria in pheochromocytoma.

## 4. Conclusions

We report a rare presentation of pheochromocytoma with heavy proteinuria in a pediatric patient with a single kidney. Pheochromocytoma is a rare etiology of hypertension, making the diagnosis particularly challenging, and its treatment requires special consideration given the potential for severe complications. The presence of nephrotic‐range proteinuria in patients with hypertension typically suggests a glomerular disease, further complicating the diagnosis of pheochromocytoma. Medical practitioners should maintain a high index of suspicion for pheochromocytoma in hypertensive patients, even when significant proteinuria is present. The mechanism of proteinuria in these cases seems to be related to glomerular hyperfiltration secondary to sustained hypertension, but immunological pathogenesis is also possible. Proteinuria in our case, and in other limited number of case reports, resolved following pheochromocytoma resection and hypertension resolution. This suggests that an early kidney biopsy to evaluate nephrotic range proteinuria may be avoidable in the setting of potential pheochromocytoma.

## Author Contributions

Jacob Miller and Nycole Hidalgo contributed to the conception, data acquisition/analysis, and writing of the manuscript. Maria Eleni Nikita and Teresa York contributed to the conception, data analysis, and revision of the manuscript. Bakri Alzarka contributed to the conception, data analysis, writing, and revision of the manuscript.

## Funding

No funding was received for this study.

## Ethics Statement

Any identifiable information has been removed from the manuscript.

## Consent

All the patients allowed personal data processing, and informed consent was obtained from all individual participants included in the study.

## Conflicts of Interest

The authors declare no conflicts of interest.

## Supporting Information

Additional supporting information can be found online in the Supporting Information section.

## Supporting information


**Supporting Information** CARE‐checklist_Pheochromocytoma case report.

## Data Availability

Data sharing is not applicable to this article as no datasets were generated or analyzed during the current study.
