# Supplementary material for: Proteinuria as an Unusual Presentation of Pheochromocytoma in Pediatric Patient With Single Kidney
Source: Case Rep Pediatr. 2026 Sep 24;2026:1761397. doi: 10.1155/crpe/1761397 (PMC13602349; doi:10.1155/crpe/1761397)
Supplement: Supplementary file 1 [file CRPE-2026-1761397-s001.pdf]

CARE Checklist of information to include when writing a case report

CARE case report guidelines

(cc) BY-NC-ND

Topic	Item	Checklist item description	Reported on Line
Title	1	The diagnosis or intervention of primary focus followed by the words "case report"	1
Key Words	2	2 to 5 key words that identify diagnoses or interventions in this case report, including "case report"	16
Abstract (no references)	3a	Introduction: What is unique about this case and what does it add to the scientific literature?	41
	3b	Main symptoms and/or important clinical findings	38
	3c	The main diagnoses, therapeutic interventions, and outcomes	40
	3d	Conclusion—What is the main "take-away" lesson(s) from this case?	43
Introduction	4	One or two paragraphs summarizing why this case is unique (**may include references**)	59
Patient Information	5a	De-identified patient specific information.	64
	5b	Primary concerns and symptoms of the patient.	64
	5c	Medical, family, and psycho-social history including relevant genetic information	67-71
	5d	Relevant past interventions with outcomes	68
Clinical Findings	6	Describe significant physical examination (PE) and important clinical findings.	74
Timeline	7	Historical and current information from this episode of care organized as a timeline	64-76
Diagnostic Assessment	8a	Diagnostic testing (such as PE, laboratory testing, imaging, surveys).	77-111
	8b	Diagnostic challenges (such as access to testing, financial, or cultural)	115
	8c	Diagnosis (including other diagnoses considered)	104,126
	8d	Prognosis (such as staging in oncology) where applicable	128-131
Therapeutic Intervention	9a	Types of therapeutic intervention (such as pharmacologic, surgical, preventive, self-care)	117-121,125
	9b	Administration of therapeutic intervention (such as dosage, strength, duration)	117-122
	9c	Changes in therapeutic intervention (with rationale)	117-122
Follow-up and Outcomes	10a	Clinician and patient-assessed outcomes (if available)	127-129
	10b	Important follow-up diagnostic and other test results	237
	10c	Intervention adherence and tolerability (How was this assessed?)	127-131
	10d	Adverse and unanticipated events	N/A
Discussion	11a	A scientific discussion of the strengths AND limitations associated with this case report	130,144,211-217
	11b	Discussion of the relevant medical literature **with references**.	161-172,179-190,200
	11c	The scientific rationale for any conclusions (including assessment of possible causes)	169-172,176,182
	11d	The primary "take-away" lessons of this case report (without references) in a one paragraph conclusion	220
Patient Perspective	12	The patient should share their perspective in one to two paragraphs on the treatment(s) they received	N/A
Informed Consent	13	Did the patient give informed consent? Please provide if requested	Yes ☑ No ☐
